# Mortality Among US Veterans Admitted to Community vs Veterans Health Administration Hospitals for COVID-19

**DOI:** 10.1001/jamanetworkopen.2023.15902

**Published:** 2023-05-30

**Authors:** Michael E. Ohl, Kelly Richardson Miell, Brice F. Beck, Bradley Mecham, George Bailey, Michelle Mengeling, Mary Vaughan-Sarrazin

**Affiliations:** 1 Veterans Rural Health Resource Center–Iowa City, Iowa City Veterans Affairs Health Care System, Iowa City, Iowa; 2 Center for Access & Delivery Research and Evaluation (CADRE), Iowa City Veterans Affairs Health Care System, Iowa City, Iowa; 3 Department of Internal Medicine, University of Iowa Carver College of Medicine, Iowa City

## Abstract

**Importance:**

Veterans Health Administration (VHA) enrollees receive care for COVID-19 in both VHA and non-VHA (ie, community) hospitals, but little is known about the frequency or outcomes of care for veterans with COVID-19 in VHA vs community hospitals.

**Objective:**

To compare outcomes among veterans admitted for COVID-19 in VHA vs community hospitals.

**Design, Setting, and Participants:**

This retrospective cohort study used VHA and Medicare data from March 1, 2020, to December 31, 2021, on hospitalizations for COVID-19 in 121 VHA and 4369 community hospitals in the US among a national cohort of veterans (aged ≥65 years) enrolled in both the VHA and Medicare with VHA care in the year prior to hospitalization for COVID-19 based on the primary diagnosis code.

**Exposure:**

Admission to VHA vs community hospitals.

**Main Outcomes and Measures:**

The main outcomes were 30-day mortality and 30-day readmission. Inverse probability of treatment weighting was used to balance observable patient characteristics (eg, demographic characteristics, comorbidity, mechanical ventilation on admission, area-level social vulnerability, distance to VHA vs community hospitals, and date of admission) between VHA and community hospitals.

**Results:**

The cohort included 64 856 veterans (mean [SD] age, 77.6 [8.0] years; 63 562 men [98.0%]) dually enrolled in the VHA and Medicare who were hospitalized for COVID-19. Most (47 821 [73.7%]) were admitted to community hospitals (36 362 [56.1%] admitted to community hospitals via Medicare, 11 459 [17.7%] admitted to community hospitals reimbursed via VHA’s Care in the Community program, and 17 035 [26.3%] admitted to VHA hospitals). Admission to community hospitals was associated with higher unadjusted and risk-adjusted 30-day mortality compared with admission to VHA hospitals (crude mortality, 12 951 of 47 821 [27.1%] vs 3021 of 17 035 [17.7%]; *P* < .001; risk-adjusted odds ratio, 1.37 [95% CI, 1.21-1.55]; *P* < .001). Readmission within 30 days was less common after admission to community compared with VHA hospitals (4898 of 38 576 [12.7%] vs 2006 of 14 357 [14.0%]; risk-adjusted hazard ratio, 0.89 [95% CI, 0.86-0.92]; *P* < .001).

**Conclusions and Relevance:**

This study found that most hospitalizations for COVID-19 among VHA enrollees aged 65 years or older were in community hospitals and that veterans experienced higher mortality in community hospitals than in VHA hospitals. The VHA must understand the sources of the mortality difference to plan care for VHA enrollees during future COVID-19 surges and the next pandemic.

## Key Points

**Question:**

Do veterans with COVID-19 experience similar outcomes when hospitalized in Veterans Health Administration (VHA) hospitals vs community hospitals?

**Findings:**

In a cohort of 64 856 VHA enrollees aged 65 years or older also enrolled in Medicare, admission to community hospitals was associated with higher risk-adjusted 30-day mortality during hospitalization for COVID-19 compared with VHA hospitals.

**Meaning:**

The findings of this study suggest that it is important for the VHA to understand sources of COVID-19 mortality differences between VHA and community hospitals to plan care for VHA enrollees during future COVID-19 surges and the next pandemic.

## Teaser

This cohort study uses Veterans Health Administration and Medicare data to compare outcomes among veterans admitted for COVID-19 in Veterans Health Administration vs community hospitals.

## Introduction

The Veterans Health Administration (VHA) is the largest integrated health care delivery system in the US, with approximately 8.7 million enrollees in 2020.^1^ Veterans Health Administration enrollees often receive care from non-VHA clinicians in the community through Medicare or other insurance providers.^2,3^ More than 90% of VHA enrollees aged 65 years or older are also enrolled in Medicare.^4^ In addition, the VHA has recently expanded efforts to pay community heath care systems to care for VHA enrollees through the Care in the Community (CITC) program.^5^ Available evidence indicates that veterans experience similar outcomes in VHA and community health care settings,^6,7,8,9,10,11,12,13^ although a recent study found better outcomes in VHA health care settings.^14^

The COVID-19 pandemic has created large surges in demand for acute care in hospitals.^15^ The VHA operates 123 acute care hospitals in the US capable of providing inpatient care for patients with severe COVID-19, but many VHA enrollees have poor geographic access to these hospitals. More than one-third (41%) of VHA enrollees aged 65 years or older—a group at risk for severe COVID-19—live more than a 60-minute drive to the nearest acute care VHA hospital (M.E.O., unpublished data, January 3, 2023). In contrast, nearly all VHA enrollees aged 65 years or older (98%) live within a 60-minute drive to 1 of the approximately 4400 community hospitals with acute care units in the US. Poor geographic access to VHA hospitals means that community hospitals have likely played a large role in caring for acutely ill veterans with severe COVID-19, but little is known about the frequency or outcomes of care in VHA vs community hospitals among veterans with COVID-19. It is important for the VHA to understand the locations and outcomes of care for veterans with COVID-19 to inform plans to deliver accessible and high-quality care for veterans during future COVID-19 case surges and the next pandemic.

We combined VHA, Medicare, and American Hospital Association survey data to describe the locations and outcomes of hospitalization for COVID-19 among VHA enrollees aged 65 years or older, including admissions to VHA hospitals, community hospitals through fee-for-service (FFS) Medicare, and community hospitals through the VHA’s CITC program. We aimed to describe the characteristics of VHA and community hospitals delivering care for veterans with COVID-19 and to compare mortality and readmission rates in VHA vs community hospitals.

## Methods

This was a retrospective cohort study of veterans aged 65 years or older enrolled in both the VHA and Medicare with a first admission or observation stay for COVID-19 in acute care settings in VHA or community hospitals between March 1, 2020, and December 31, 2021. Reporting followed the Strengthening the Reporting of Observational Studies in Epidemiology (STROBE) reporting guideline.^16^ The institutional review board at The University of Iowa approved all analyses and granted a waiver of informed consent because the data were deidentified.

### Data Sources and Patient Cohort

We obtained data from 5 sources: (1) the VHA Corporate Data Warehouse, which provided data on VHA enrollee demographic characteristics (eg, sex, age, race and ethnicity, and residential address), date of death within or outside of hospitals, and care in VHA facilities including acute care stays, outpatient visits, and diagnoses by the *International Statistical Classification of Diseases and Related Health Problems, Tenth Revision, Clinical Modification* (*ICD-10-CM*); (2) the VHA Program Integrity Tool, which provided data on acute care stays in community hospitals reimbursed by VHA through the CITC program, including hospital name, dates of stay, and diagnosis codes; (3) the Veterans Affairs Information Resource Center, which provided Centers for Medicare & Medicaid Systems data on Medicare enrollment, mortality, and FFS Medicare claims among VHA enrollees, including outpatient and inpatient claims^17^; (4) the American Hospital Association 2020 survey of US hospitals, which provided data on characteristics of VHA and community hospitals in the US^18^; and (5) the Agency for Toxic Substances and Disease Registry of the Centers for Disease Control and Prevention, which provided data on the Social Vulnerability Index, an area measure of socioeconomic disadvantage and vulnerability to infectious disease outbreaks for census tracts where veterans resided.^19^ We did not have data on Medicare claims for veterans enrolled in Managed Medicare (Medicare Advantage) programs.

We began by identifying all hospital admissions or observation stays with a primary diagnosis of COVID-19 (*ICD-10-CM* code U07.1) in VHA or community hospitals. We created hospital episodes linked by patient transfers by joining hospital stays with admission and discharge times within 24 hours and classified the admitting hospital based on the final site of care. For example, a hospitalization that began in a community hospital via Medicare reimbursement with patient transfer to a VHA hospital was classified as a VHA stay with a variable indicating transfer.

We then created an analytic cohort to compare patient outcomes in VHA and community hospitals (Figure). To reduce selection bias in comparisons and facilitate risk adjustment, this cohort excluded (1) patients with no VHA care in the year prior to admission, to ensure availability of risk adjustment variables based on prior care at time of admission (35% excluded); (2) patients not enrolled in FFS Medicare in the 60 days prior to admission, because these patients were not eligible for cohort inclusion based on admission to community hospitals via Medicare (11% excluded); (3) patients admitted to hospitals that we were not able to match to the American Hospital Association survey (2% excluded); and (4) patients missing risk-adjustment variables (0.4% excluded). The analytic cohort included only first admissions for COVID-19, excluding readmissions (n = 64 856; Figure). In analyses of 30-day hospital readmissions, we further excluded patients who died during the initial hospitalization.

**Figure.**
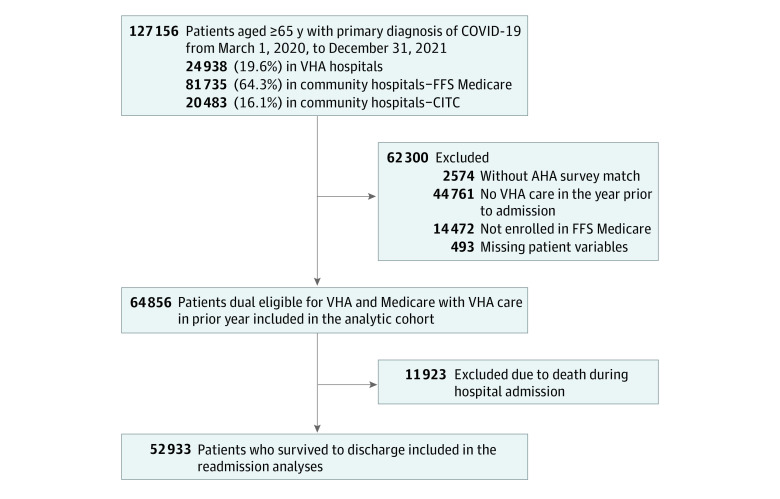
Cohort Derivation Flowchart AHA indicates American Hospital Association; CITC, Care in the Community; FFS, fee-for-service; and VHA, Veterans Health Administration.

### Variables

The primary exposure variable was an indicator of admission to a VHA or community hospital (ie, combining community hospitalizations via Medicare and the VHA’s CITC program). We also conducted secondary analyses that examined outcomes separately by admission to community hospital via Medicare or via the VHA’s CITC program. Outcomes were mortality within 30 days of admission among all patients and time to readmission to either a VHA or community hospital within 30 days of discharge. Patient-level descriptive and risk-adjustment variables included (1) age; (2) sex; (3) race and ethnicity; (4) residence in urban, rural, or highly rural environments based on the measure used by VHA^20,21^; (5) comorbidities based on inpatient and outpatient *ICD-10-CM* codes in VHA and Medicare in the year prior to admission using the method of Quan et al^22^; (6) a summary comorbidity index using the method of Gagne et al^23^; (7) mechanical ventilation on day of admission based on *ICD-10-CM* procedure codes (0BH17EZ, 0BH18EZ, 5A1935Z, 5A1945Z, and 5A1955Z) as a measure of illness severity at admission; (8) an indicator of residence in a census tract of high social vulnerability (ie, residence in top 10% most vulnerable census tracts based on the Centers for Disease Control and Prevention Social Vulnerability Index); (9) straight-line distance from patient residence to nearest VHA and community acute care hospital; (10) an indicator of transfer from another hospital; and (11) date of admission in 6-month increments (ie, March to August 2020, September 2020 to February 2021, March to August 2021, and September to December 2021). We included race and ethnicity as self-reported by VHA enrollees and recorded in administrative data to identify possible differences in locations and outcomes of hospitalization.

Hospital-level variables included urban vs rural hospital location, number of acute care medical and surgical beds, number of intensive care unit beds, teaching status based on Council of Teaching Hospitals and Health Systems membership, academic affiliation based on medical school affiliation reported to the American Medical Association, and Critical Access Hospital designation.^24^ Critical Access Hospital designation was relevant only for community hospitals.

### Statistical Analysis

We began by comparing the characteristics of patients admitted to VHA vs community hospitals. We also compared the characteristics of admitting VHA vs community hospitals using the hospital as the unit of analysis. Comparisons used χ^2^ tests for categorical variables and rank sum tests for continuous variables. All tests were 2-sided, and we defined significance based on *P* < .01 because of multiple comparisons.

Our primary analysis compared 30-day mortality and 30-day readmission between patients admitted to VHA hospitals and patients admitted to community hospitals. Secondary analysis separated community admissions paid by Medicare or the VHA’s CITC program. We used inverse probability of treatment weighting (IPTW) to control for differences in the characteristics of patients admitted to VHA hospitals vs those admitted to community hospitals, and we estimated the mean treatment effects associated with VHA vs community hospitalization. In the primary analysis for 30-day mortality, the propensity for admission to a VHA hospital (rather than a community hospital) was estimated using logistic regression, in which the dependent variable was hospitalization in the VHA hospital and the independent variables included patient age, race and ethnicity, sex, rural residence, Social Vulnerability Index, date of admission, distance to nearest VHA hospital, distance to nearest community hospital, comorbidities, and acuity (measured as need for mechanical ventilation on date of admission). To address outlying and nonoverlapping treatment weights between groups, we examined the distribution of propensities for VHA and community-based hospitalizations and excluded patients whose propensity for VHA admission exceeded minimum and maximum cutoffs for inclusion, in which the minimum cutoff was defined as the first percentile propensity score for either VHA or community-based admissions (whichever was larger), and the maximum cutoff was defined as the 99th percentile propensity score for either VHA or community-based admissions (whichever was smaller; eFigure in Supplement 1). Inverse probability of treatment weights were then calculated for the remaining patients as the inverse of the probability of exposure (ie, community vs VHA hospital admission). We calculated standardized differences to verify comparability of characteristics of patients admitted to VHA vs community hospitals after weighting.

Risk-adjusted 30-day mortality was then compared for VHA and community hospitalizations using IPTW in generalized estimating equations with a binomial distribution and logit link and a significance threshold of *P* < .01. Models accounted for clustering of patients within hospitals using an exchangeable working correlation matrix. Propensity models, IPTW, and final generalized estimating equation models were reestimated to evaluate differences in 30-day readmission between VHA and community hospitals, after excluding 11 923 patients who died during the index hospitalization. We calculated cause-specific hazard ratios (HRs) for associations between admitting hospital type and time to readmission within 30 days, accounting for competing risk of out-of-hospital death after discharge. Secondary analyses followed a similar approach, except propensities for admission to a VHA hospital, community hospital paid by Medicare, or community hospital paid by the VHA’s CITC program were estimated using a multinomial logit model. All analyses used SAS software, version 9.4 (SAS Institute Inc).

## Results

We identified 127 156 hospitalizations with a principal diagnosis of COVID-19 among VHA enrollees aged 65 years or older between March 1, 2020, and December 31, 2021 (eTable 1 in Supplement 1). Hospitalized patients excluded from analyses due to lack of dual enrollment in Medicare or VHA care in the prior year had similar 30-day mortality compared with the included patients, both overall and when stratified by VHA and community hospital admission (eTable 2 in Supplement 1). The analytic cohort of 64 856 veterans (mean [SD] age, 77.6 [8.0] years; 63 562 men [98.0%]) enrolled in both the VHA and Medicare with VHA care in the prior year included 17 035 (26.3%) admitted to VHA hospitals, 36 362 (56.1%) admitted to community hospitals via FFS Medicare, and 11 459 (17.7%) admitted to community hospitals via the VHA’s CITC program (Table 1). Compared with VHA enrollees admitted to VHA hospitals, VHA enrollees admitted to community hospitals were older (mean [SD] age, 78.2 [8.0] vs 75.8 [7.9] years; *P* < .001), more likely to be White (39 271 of 47 821 [82.1%] vs 11 746 of 17 035 [69.0%]; *P* < .001), less likely to live in urban areas (25 960 of 47 821 [54.3%] vs11 403 of 17 035 [66.9%]; *P* < .001), and less likely to live in census tracts with high social vulnerability (3068 of 47 821 [6.4%] vs 1762 of 17 035 [10.3%]; *P* < .001). Several age-related comorbidities were more common among patients admitted to community hospitals than to VHA hospitals (congestive heart failure, 15 299 of 47 821 [32.0%] vs 4730 of 17 035 [27.8%]; *P* < .001; stroke, 11 785 of 47 821 [24.6%] vs 3410 of 17 035 [20.0%]; *P* < .001; and kidney disease, 17 359 of 47 821 [36.3%] vs 5382 of 17 035 [31.6%]; *P* < .001). In contrast, alcohol use conditions were somewhat more common among patients in VHA hospitals (1368 of 17 035 [8.0%] vs 2464 of 47 821 [5.2%]; *P* < .001). The median distance to the nearest VHA hospital was 113.6 km (IQR, 115.9-187.0 km) among enrollees admitted to community hospitals vs 36.2 km (IQR, 16.3-85.6 km) among those admitted to VHA hospitals (*P* < .001).

**Table 1.** Characteristics of Veterans Dually Enrolled in VHA and Medicare Hospitalized for COVID-19, by Hospital Type and Payment Source

Characteristic	Hospital type			Payment source		
	VHA hospital (n = 17 035 [26.3%])	Any community hospital (n = 47 821 [73.7%])	*P* value	Community hospital via Medicare (n = 36 362 [56.1%])	Community hospital via CITC (n = 11 459 [17.7%])	*P* value
Age, mean (SD), y	75.8 (7.9)	78.2 (8.0)	<.001	79.0 (8.1)	75.7 (7.1)	<.001
Age group, No. (%), y						
65-69	3169 (18.6)	5618 (11.8)	<.001	3772 (10.4)	1846 (16.1)	<.001
70-74	6018 (35.3)	14 297 (29.9)		9856 (27.1)	4441 (38.8)	
75-79	3425 (20.1)	9552 (20.0)		7152 (19.7)	2400 (20.9)	
80-84	1807 (10.6)	6572 (13.7)		5388 (14.8)	1184 (10.3)	
85-90	1468 (8.6)	6657 (13.9)		5690 (15.7)	967 (8.4)	
>90	1148 (6.7)	5125 (10.7)		4504 (12.4)	621 (5.4)	
Sex, No. (%)						
Male	16 611 (97.5)	46 951 (98.2)	<.001	35 716 (98.2)	11 235 (98.0)	.21
Female	424 (2.5)	870 (1.8)		646 (1.8)	224 (2.0)	
Race and ethnicity, No. (%)						
Black, not Hispanic	3595 (21.1)	5196 (10.9)	<.001	4147 (11.4)	1049 (9.2)	<.001
Hispanic	1249 (7.3)	2100 (4.4)		1545 (4.3)	555 (4.8)	
White, not Hispanic	11 746 (69.0)	39 271 (82.1)		29 727 (81.8)	9544 (83.3)	
Other^a^	440 (2.6)	1230 (2.6)		922 (2.5)	308 (2.7)	
Unknown or missing	5 (0.03)	24 (0.1)		21 (0.1)	3 (0.03)	
Residence, No. (%)						
Urban	11 403 (66.9)	25 960 (54.3)	<.001	20 699 (56.9)	5261 (45.9)	<.001
Rural	4960 (29.1)	18 769 (39.3)		13 533 (37.2)	5236 (45.7)	
Highly rural	672 (3.9)	3089 (6.4)		2127 (5.9)	962 (8.4)	
High social vulnerability census tract, No. (%)	1762 (10.3)	3068 (6.4)	<.001	2287 (6.3)	781 (6.8)	.05
Admission month, No. (%)						
March-August 2020	2858 (16.7)	6581 (13.8)	<.001	5571 (15.3)	1010 (8.8)	<.001
September 2020-February 2021	7890 (46.3)	24 632 (51.5)		19 346 (53.2)	5286 (46.1)	
March-August 2021	2607 (15.3)	7052 (14.8)		5024 (13.8)	2028 (17.7)	
September-December 2021	3680 (21.6)	9556 (20.0)		6421 (17.7)	3135 (27.4)	
Gagne comorbidity index, median (IQR)	5.0 (2.0-8.0)	5.0 (3.0-8.0)	.001	5.0 (3.0-8.0)	4.0 (2.0-7.0)	<.001
Comorbidities, No. (%)						
Hypertension	14 053 (82.4)	41 446 (86.7)	<.001	32 193 (88.5)	9253 (80.8)	<.001
Congestive heart failure	4730 (27.8)	15 299 (32.0)	<.001	12 192 (33.5)	3107 (27.1)	<.001
Arrhythmia	7061 (41.5)	21 851 (45.7)	<.001	17 575 (48.3)	4276 (37.3)	<.001
Myocardial infarction	2217 (13.0)	7595 (15.9)	<.001	5998 (16.5)	1597 (13.9)	<.001
Chronic lung disease	6453 (37.9)	19 063 (39.9)	<.001	14 559 (40.0)	4505 (39.3)	.16
Obesity	4197 (24.6)	13 309 (27.8)	<.001	10 268 (28.2)	3041 (26.5)	<.001
Stroke	3410 (20.0)	11 785 (24.6)	<.001	9662 (26.6)	2123 (18.5)	<.001
Dementia	2970 (17.4)	8061 (16.9)	.08	6565 (18.1)	1496 (13.1)	<.001
Liver disease	1667 (9.8)	4113 (8.6)	<.001	3247 (8.9)	866 (7.6)	<.001
Diabetes	8891 (52.2)	24 779 (51.8)	.40	18 918 (52.0)	5861 (51.2)	<.001
Cancer	3315 (19.5)	9814 (20.5)	.003	7916 (21.8)	1898 (16.6)	<.001
Kidney disease	5382 (31.6)	17 359 (36.3)	<.001	14 019 (38.6)	3340 (29.2)	<.001
Depression	5101 (29.9)	13 355 (27.9)	<.001	10 209 (28.1)	3146 (27.5)	.20
Alcohol use condition	1368 (8.0)	2464 (5.2)	<.001	1716 (4.7)	748 (6.5)	<.001
Drug use condition	820 (4.8)	1603 (3.4)	<.001	1176 (3.2)	427 (3.7)	.001
Transfer in, No. (%)	1204 (7.1)	5325 (11.1)	<.001	4078 (11.2)	1247 (10.9)	.32
Mechanical ventilation on admission, No. (%)	550 (3.2)	1516 (3.2)	.71	1167 (3.2)	349 (3.1)	.39
Distance to nearest VHA hospital, median (IQR), km	36.2 (16.3-85.6)	113.6 (115.9-187.0)	<.001	106.4 (46.5-180.7)	132.0 (72.4-209.1)	<.001
Distance to nearest community hospital, median (IQR), km	9.2 (4.8-17.9)	10.3 (5.0-20.6)	<.001	10.0 (4.8-19.6)	11.3 (5.2-23.7)	<.001
30-d Mortality	3021 (17.7)	12 951 (27.1)	<.001	10 111 (27.8)	2840 (24.8)	<.001
30-d Readmission^b^	2006/14 357 (14.0)	4898/38 576 (12.7)	<.001	3636/29 204 (12.5)	1262/9372 (13.5)	<.001

Abbreviations: CITC, Care in the Community; VHA, Veterans Health Administration.

^a^ American Indian, Asian, or multiracial.

^b^ Readmission applies to the 52 933 patients who survived to discharge.

Compared with VHA enrollees admitted to community hospitals via Medicare, enrollees admitted to community hospitals via the VHA’s CITC program were younger (mean [SD] age, 75.7 [7.1] vs 79.0 [8.1] years; *P* < .001), less likely to live in urban areas (5261 of 11 459 [45.9%] vs 20 699 of 36 362 [56.9%]; *P* < .001), and lived farther from the nearest VHA hospital (median distance, 132.0 km [IQR, 72.4-209.1 km] vs 106.4 km [IQR, 46.5-180.7 km]; *P* < .001) (Table 1). Admissions via the VHA’s CITC program became more common with time; 27.4% of CITC admissions (3135 of 11 459) were in the last 4 months of cohort compared with 17.7% of Medicare admissions (6421 of 36 362) (*P* < .001). The characteristics of the 52 933 patients who survived to discharge and were included in readmission analyses are included in eTable 3 in Supplement 1.

Veterans Health Administration enrollees in the analytic cohort received care for COVID-19 in 121 VHA hospitals and 4369 community hospitals (Table 2). Using the hospital as the unit of analysis, this study found that community hospitals caring for veterans with COVID-19 were less likely than VHA hospitals to be in an urban area (2352 [53.8%] vs 108 [89.3%]; *P* < .001) and had fewer total acute care medical and surgical beds (mean [SD], 170.9 [211.0] vs 253.6 [217.1]; *P* < .001). Community hospitals were less likely than VHA hospitals to be members of the Council of Teaching Hospitals (234 [5.4%] vs 26 [21.5%]; *P* < .001) or academically affiliated (1563 [35.8%] vs 103 [85.1%]; *P* < .001). Approximately one-quarter of community hospitals caring for VHA enrollees with COVID-19 (1079 [24.7%]) were rural hospitals designated as Critical Access Hospitals. Most community hospitals (2734 of 4369 [62.6%]) admitted VHA enrollees via both Medicare and the VHA’s CITC program, and characteristics were similar for hospitals with or without CITC admissions.

**Table 2.** Characteristics of Admitting Hospitals, by Hospital Type

Characteristic	VHA hospital (n=121)	Community hospital (n=4369)	*P* value
Total beds, mean (SD)	253.6 (217.1)	170.9 (211.0)	<.001
ICU beds, mean (SD)	2.9 (6.9)	10.6 (18.4)	<.001
Teaching hospital, No. (%)	26 (21.5)	234 (5.4)	<.001
Academic affiliation, No. (%)	103 (85.1)	1563 (35.8)	<.001
Location, No. (%)			
Urban	108 (89.3)	2352 (53.8)	<.001
Rural	13 (10.7)	1664 (38.1)	
Highly rural	0	351 (8.0)	
Critical Access Hospital, No. (%)	NA	1079 (24.7)	NA

Abbreviations: ICU, intensive care unit; NA, not applicable; VHA, Veterans Health Administration.

In the multivariable logistic regression model used to estimate weights for IPTW analyses, admission to a VHA vs a community hospital was associated with younger patient age, shorter distance to a VHA hospital, residence in a high social vulnerability census tract, and higher Gagne comorbidity score (eTable 4 in Supplement 1). Patient characteristics in VHA vs community hospitals were well balanced after applying IPTW (ie, all standardized differences were <7% in the IPTW cohorts; eTable 5 in Supplement 1).

Veterans Health Administration enrollees experienced higher unadjusted mortality after admission to community hospitals compared with VHA hospitals (12 951 of 47 821 [27.1%] vs 3021 of 17 035 [17.7%]; *P* < .001) (Table 1). In analyses using IPTW in the trimmed cohort, mortality was substantially higher in community compared with VHA hospitals both before weighting (unadjusted odds ratio [OR], 1.76 [95% CI, 1.64-1.90]; *P* < .001) (Table 3) and after adjustment for patient characteristics (risk-adjusted OR, 1.37 [95% CI, 1.21-1.55]; *P* < .001) (Table 3). Readmission within 30 days was less common after admission to community hospitals compared with VHA hospitals (4898 of 38 576 [12.7%] vs 2006 of 14 357 [14.0%]) in unadjusted (HR, 0.90 [95% CI, 0.84-0.97]; *P* = .005) (Table 3) and risk-adjusted analyses (HR, 0.89 [95% CI, 0.86-0.92]; *P* < .001) (Table 3). Mortality and readmission outcomes were similar after admission to community hospitals via Medicare vs via the VHA’s CITC program compared with VHA hospitals (Table 3).

**Table 3.** Associations Between Admitting Hospital and Care Outcomes

Analysis	30-d Mortality		30-d Readmission	
	Odds ratio (95% CI)	*P* value	Hazard ratio (95% CI)	*P* value
**Primary analysis: VHA vs community hospital**				
Unadjusted				
VHA hospital	1 [Reference]	NA	1 [Reference]	NA
Community hospital	1.76 (1.64-1.90)	<.001	0.90 (0.84-0.97)	.005
Risk-adjusted^a^				
VHA hospital	1 [Reference]	NA	1 [Reference]	NA
Community hospital	1.37 (1.21-1.55)	<.001	0.89 (0.86-0.92)	<.001
**Secondary analysis: VHA vs community hospital–Medicare vs community hospital–CITC**				
Unadjusted				
VHA hospital	1 [Reference]	NA	1 [Reference]	NA
Community hospital: Medicare	1.83 (1.70-1.98)	<.001	0.88 (0.82-0.95)	<.001
Community hospital: CITC	1.55 (1.42-1.69)	<.001	0.97 (0.88-1.06)	.45
Risk-adjusted^a^				
VHA hospital	1 [Reference]	NA	1 [Reference]	NA
Community hospital: Medicare	1.35 (1.20-1.53)	<.001	0.82 (0.74-0.91)	<.001
Community hospital: CITC	1.44 (1.26-1.64)	<.001	0.91 (0.81-1.01)	.12

Abbreviations: CITC, Care in the Community; NA, not applicable; VHA, Veterans Health Administration.

^a^ Adjusted for patient characteristics (ie, demographic characteristics, comorbidity, area-level social vulnerability, mechanical ventilation on admission, transfer in, rural residence, distance to nearest VHA and community hospital) using inverse probability of treatment weighting.

## Discussion

In this national study of VHA enrollees aged 65 years or older also enrolled in Medicare, most hospitalizations for COVID-19 were in community hospitals, with VHA hospitals playing a smaller role. Veterans experienced substantially higher risk-adjusted mortality in community hospitals than in VHA hospitals, while readmission was more common after VHA hospitalization. These findings are important for veterans and VHA leaders working to plan optimal care during future COVID-19 case surges and the next pandemic, in particular in the context of VHA efforts to expand programs to pay community health care systems to care for veterans.

Our findings must be considered in the context of prior studies comparing the quality and outcomes of health care in VHA and non-VHA settings.^6,7,8,9,10,11,12,13,14^ Studies of care processes have generally found that the quality of VHA care compares favorably with non-VHA care, but studies of care outcomes have been mixed.^10^ Most prior studies compared outcomes for veterans receiving care in VHA facilities with outcomes for nonveterans receiving care in non-VHA facilities; these studies may have been biased if VHA enrollees tended to have worse health than the general population, as has been previously reported.^25^ A recent study of VHA enrollees with acute conditions transported by ambulance found lower mortality among those transported to VHA hospitals compared with those transported to non-VHA hospitals.^14^

There are several potential explanations for our finding of higher COVID-19 mortality in community hospitals compared with VHA hospitals. As in all observational studies, there is potential for unadjusted confounding associated with differences in health status prior to COVID-19 infection or in illness severity at time of admission. It is also possible that the quality of care differed in VHA and community hospital settings. Since the beginning of the COVID-19 pandemic, the VHA health care system has worked to rapidly implement advances in care according to the latest treatment guidelines, including use of antiviral medications, corticosteroids, and other anti-inflammatory medications for people with severe COVID-19.^26,27^

In contrast to mortality, readmission rates were somewhat higher after admission to VHA hospitals compared with community hospitals. This difference was not sensitive to adjustment for patient characteristics, suggesting that higher readmission rates after admission to VHA hospitals may reflect a feature of the VHA care system and not case mix. Other studies have reported higher readmission rates in VHA hospitals compared with community hospitals.^9^ Higher readmission rates may partly result from VHA programs to track veterans and manage care transitions after hospital discharge.^28^ Future studies should assess whether higher readmission rates in VHA hospitals reflect an undesired outcome or a necessary aspect of efforts to improve access to primary care during care transitions.

Compared with VHA hospitals, community hospitals played a disproportionate role in care for rural veterans with COVID-19, probably because of poor geographic access to VHA hospitals among rural veterans. Nearly half the community hospitals (46.1%) caring for VHA enrollees with COVID-19 were in rural areas, and 24.7% were Critical Access Hospitals, a designation granted to small, rural hospitals to maintain their financial viability and preserve access to care in rural communities.^24^ Rural hospitals are financially stressed and closing at high rates.^29,30^ It is important for the VHA to understand the role of rural community hospitals in acute care for rural VHA enrollees—both during surges in demand for care during pandemics and overall—so that the VHA can support and collaborate with these hospitals to maintain access to care for rural veterans.

### Limitations

This study has some limitations. We identified patients with COVID-19 using the *ICD-10-CM* code U07.1,^31^ and it is possible this code misclassified patients. However, available evidence from both VHA and non-VHA settings indicates that this *ICD-10-CM* code has high specificity and a positive predictive value of more than 90% in the inpatient setting.^32,33,34,35^ We lacked detailed data on illness severity at admission based on vital signs, laboratory test results, or oxygen requirements, and we did not have data on illness severity on day of discharge for risk adjustment in readmission analyses. We did not include data on COVID-19 vaccine receipt prior to admission as a potential confounder because most patients in our study were admitted before the vaccine was widely available. We were also concerned that vaccine receipt in community settings was not reliably recorded in any of the data sets available to us, and the undercounting of veterns receiving the vaccine outside VHA facilities could have been differential among VHA enrollees admitted to VHA and community hospitals. Additionally, we lacked data on community hospital admissions paid for through Managed Medicare (Medicare Advantage) or private insurance and therefore may have underestimated the role of community hospitals.

## Conclusions

This cohort study found that most hospitalizations for COVID-19 among VHA enrollees aged 65 years or older were in community hospitals and that veterans experienced higher mortality in community hospitals than in VHA hospitals. The VHA must understand the sources of the mortality difference to plan care for VHA enrollees during future COVID-19 surges and the next pandemic.

## Notes

Supplement 1. **eTable 1.** Characteristics of 127,156 VHA Enrollees Age 65+ Hospitalized for COVID-19 Between March 2020 and December 2021 Before Exclusions, by Hospital Type and Payer **eTable 2.** Characteristics of Excluded and Included Patients **eTable 3.** Characteristics of 52,933 Patients Surviving to Hospital Discharge and Included in Readmission Analyses, by Hospital Type **eTable 4.** Multivariable Logistic Regression to Estimate Propensity for VHA Hospital Admission (Odds Ratios for VHA Admission Referent to Community) **eFigure.** Distributions of Propensity for VHA Admission (i.e., Treatment Weights) Among Patients Admitted to VHA and Community Hospitals and Approach to Trimming of Weights (Shaded Area Included in Analyses Following Trimming of Outlying Weights, See Text) **eTable 5.** Characteristics of VHA-Medicare Dual Enrollees Age 65+ Hospitalized for COVID-19, by Hospital Admission in VHA vs Community, Before and After Inverse Probability of Treatment Weighting (IPTW)

Supplement 2.
Data Sharing Statement

## References

[1] US Department of Veterans Affairs. About VHA. Accessed December 30, 2022. https://www.va.gov/health/aboutvha.asp

[2] Hynes DM, Koelling K, Stroupe K, . Veterans’ access to and use of Medicare and Veterans Affairs health care. Med Care. 2007;45(3):214-223. doi:10.1097/01.mlr.0000244657.90074.b7 17304078

[3] West AN, Charlton ME, Vaughan-Sarrazin M. Dual use of VA and non-VA hospitals by veterans with multiple hospitalizations. BMC Health Serv Res. 2015;15:431. doi:10.1186/s12913-015-1069-8 26416176 PMC4587652

[4] US Department of Veterans Affairs. Chief Strategy Office. Accessed November 9, 2020. https://www.va.gov/HEALTHPOLICYPLANNING/SOE2018/2018EnrolleeDataFindingsReport_9January2019Final508Compliant.pdf

[5] 115th US Congress. VA Mission Act of 2018. Accessed November 11, 2020. https://www.congress.gov/bill/115th-congress/senate-bill/2372/text

[6] Anhang Price R, Sloss EM, Cefalu M, Farmer CM, Hussey PS. Comparing quality of care in Veterans Affairs and non–Veterans Affairs settings. J Gen Intern Med. 2018;33(10):1631-1638. doi:10.1007/s11606-018-4433-7 29696561 PMC6153237

[7] Asch SM, McGlynn EA, Hogan MM, . Comparison of quality of care for patients in the Veterans Health Administration and patients in a national sample. Ann Intern Med. 2004;141(12):938-945. doi:10.7326/0003-4819-141-12-200412210-00010 15611491

[8] Henderson WG, Khuri SF, Mosca C, Fink AS, Hutter MM, Neumayer LA. Comparison of risk-adjusted 30-day postoperative mortality and morbidity in Department of Veterans Affairs hospitals and selected university medical centers: general surgical operations in men. J Am Coll Surg. 2007;204(6):1103-1114. doi:10.1016/j.jamcollsurg.2007.02.068 17544069

[9] Nuti SV, Qin L, Rumsfeld JS, . Association of admission to Veterans Affairs hospitals vs non–Veterans Affairs hospitals with mortality and readmission rates among older men hospitalized with acute myocardial infarction, heart failure, or pneumonia. JAMA. 2016;315(6):582-592. doi:10.1001/jama.2016.0278 26864412 PMC5459395

[10] O’Hanlon C, Huang C, Sloss E, . Comparing VA and non-VA quality of care: a systematic review. J Gen Intern Med. 2017;32(1):105-121. doi:10.1007/s11606-016-3775-2 27422615 PMC5215146

[11] Rosenthal GE, Sarrazin MV, Harper DL, Fuehrer SM. Mortality and length of stay in a Veterans Affairs hospital and private sector hospitals serving a common market. J Gen Intern Med. 2003;18(8):601-608. doi:10.1046/j.1525-1497.2003.11209.x 12911641 PMC1494896

[12] Trivedi AN, Matula S, Miake-Lye I, Glassman PA, Shekelle P, Asch S. Systematic review: comparison of the quality of medical care in Veterans Affairs and non–Veterans Affairs settings. Med Care. 2011;49(1):76-88. doi:10.1097/MLR.0b013e3181f53575 20966778

[13] Waldo SW, Glorioso TJ, Barón AE, . Outcomes among patients undergoing elective percutaneous coronary intervention at Veterans Affairs and community care hospitals. J Am Coll Cardiol. 2020;76(9):1112-1116. doi:10.1016/j.jacc.2020.05.086 32854846

[14] Chan DC, Danesh K, Costantini S, Card D, Taylor L, Studdert DM. Mortality among US veterans after emergency visits to Veterans Affairs and other hospitals: retrospective cohort study. BMJ. 2022;376:e068099. doi:10.1136/bmj-2021-068099 35173019 PMC8848127

[15] GitHub. NYtimes / COVID-19-data. Accessed November 19, 2022. https://github.com/nytimes/covid-19-data

[16] von Elm E, Altman DG, Egger M, Pocock SJ, Gøtzsche PC, Vandenbroucke JP; STROBE Initiative. The Strengthening the Reporting of Observational Studies in Epidemiology (STROBE) statement: guidelines for reporting observational studies. Lancet. 2007;370(9596):1453-1457. doi:10.1016/S0140-6736(07)61602-X 18064739

[17] U.S. Department of Veterans Affairs. VA Information Resource Center (ViREC): VA/CMS data for research. Accessed November 12, 2020. https://www.virec.research.va.gov/VACMS/About.asp

[18] American Hospital Association. American Hospital Survey. Accessed December 30, 2022. https://www.ahasurvey.org/taker/asindex.do

[19] Centers for Disease Control and Prevention, Agency for Toxic Substances and Disease Registry. CDC/ATSDR Social Vulnerability Index. Accessed November 11, 2020. https://www.atsdr.cdc.gov/placeandhealth/svi/index.html

[20] U.S. Department of Veterans Affairs, Office of Rural Health. VHA and ORH adopt new system to define “rural.” Accessed December 30, 2022. https://www.ruralhealth.va.gov/rural-definition.asp

[21] West AN, Lee RE, Shambaugh-Miller MD, . Defining “rural” for veterans’ health care planning. J Rural Health. 2010;26(4):301-309. doi:10.1111/j.1748-0361.2010.00298.x 21029164

[22] Quan H, Sundararajan V, Halfon P, . Coding algorithms for defining comorbidities in *ICD-9-CM* and *ICD-10* administrative data. Med Care. 2005;43(11):1130-1139. doi:10.1097/01.mlr.0000182534.19832.83 16224307

[23] Gagne JJ, Glynn RJ, Avorn J, Levin R, Schneeweiss S. A combined comorbidity score predicted mortality in elderly patients better than existing scores. J Clin Epidemiol. 2011;64(7):749-759. doi:10.1016/j.jclinepi.2010.10.004 21208778 PMC3100405

[24] Centers for Medicare & Medicaid Services (CMS), Department of Health and Human Services. Medicare and Medicaid programs; reform of hospital and critical access hospital conditions of participation: final rule. Fed Regist. 2012;77(95):29034-29076. 22606738

[25] Agha Z, Lofgren RP, VanRuiswyk JV, Layde PM. Are patients at Veterans Affairs medical centers sicker? a comparative analysis of health status and medical resource use. Arch Intern Med. 2000;160(21):3252-3257. doi:10.1001/archinte.160.21.3252 11088086

[26] World Health Organization. Therapeutics and COVID-19: living guideline. Accessed December 3, 2020. https://www.who.int/publications/i/item/therapeutics-and-covid-19-living-guideline 35917393

[27] Infectious Diseases Society of America. IDSA guidelines on the treatment and management of patients with COVID-19. Updated December 2, 2020. Accessed December 3, 2020. https://www.idsociety.org/practice-guideline/covid-19-guideline-treatment-and-management/#toc-8

[28] Weinberger M, Oddone EZ, Henderson WG. Does increased access to primary care reduce hospital readmissions? Veterans Affairs Cooperative Study Group on Primary Care and Hospital Readmission. N Engl J Med. 1996;334(22):1441-1447. doi:10.1056/NEJM199605303342206 8618584

[29] Frakt AB. The rural hospital problem. JAMA. 2019;321(23):2271-2272. doi:10.1001/jama.2019.7377 31211334

[30] Kaufman BG, Thomas SR, Randolph RK, . The rising rate of rural hospital closures. J Rural Health. 2016;32(1):35-43. doi:10.1111/jrh.12128 26171848

[31] Centers for Disease Control and Prevention. *ICD-10-CM* official coding guidelines—supplement: coding encounters related to COVID-19 coronavirus outbreak. Accessed March 28, 2020. https://www.cdc.gov/nchs/data/icd/ICD-10-CM-Official-Coding-Gudance-Interim-Advice-coronavirus-feb-20-2020.pdf

[32] Bhatt AS, McElrath EE, Claggett BL, . Accuracy of ICD-10 diagnostic codes to identify COVID-19 among hospitalized patients. J Gen Intern Med. 2021;36(8):2532-2535. doi:10.1007/s11606-021-06936-w 34100236 PMC8183587

[33] Kadri SS, Gundrum J, Warner S, . Uptake and accuracy of the diagnosis code for COVID-19 among US hospitalizations. JAMA. 2020;324(24):2553-2554. doi:10.1001/jama.2020.20323 33351033 PMC7756233

[34] Kluberg SA, Hou L, Dutcher SK, . Validation of diagnosis codes to identify hospitalized COVID-19 patients in health care claims data. Pharmacoepidemiol Drug Saf. 2022;31(4):476-480. doi:10.1002/pds.5401 34913208

[35] Lynch KE, Viernes B, Gatsby E, . Positive predictive value of COVID-19 *ICD-10* diagnosis codes across calendar time and clinical setting. Clin Epidemiol. 2021;13:1011-1018. doi:10.2147/CLEP.S335621 34737645 PMC8558427

