# Supplementary material for: Mortality Among US Veterans Admitted to Community vs Veterans Health Administration Hospitals for COVID-19
Source: JAMA Netw Open. 2023 May 30;6(5):e2315902. doi: 10.1001/jamanetworkopen.2023.15902 (PMC10230320; doi:10.1001/jamanetworkopen.2023.15902)
Supplement: Supplement 1. — eTable 1. Characteristics of 127,156 VHA Enrollees Age 65+ Hospitalized for COVID-19 Between March 2020 and December 2021 Before Exclusions, by Hospital Type and Payer eTable 2. Characteristics of Excluded and Included Patients eTable 3. Characteristics of 52,933 Patients Surviving to Hospital Discharge and Included in Readmission Analyses, by Hospital Type eTable 4. Multivariable Logistic Regression to Estimate Propensity for VHA Hospital Admission (Odds Ratios for VHA Admission Referent to Community) eFigure. Distributions of Propensity for VHA Admission (i.e., Treatment Weights) Among Patients Admitted to VHA and Community Hospitals and Approach to Trimming of Weights (Shaded Area Included in Analyses Following Trimming of Outlying Weights, See Text) eTable 5. Characteristics of VHA-Medicare Dual Enrollees Age 65+ Hospitalized for COVID-19, by Hospital Admission in VHA vs Community, Before and After Inverse Probability of Treatment Weighting (IPTW) [file jamanetwopen-e2315902-s001.pdf]

Supplementary Online Content

Ohl ME, Richardson Miell K, Beck BF, et al. Mortality among US veterans admitted to community vs Veterans Health Administration hospitals for COVID-19. *JAMA Netw Open.* 2023;6(5):e2315902. doi:10.1001/jamanetworkopen.2023.15902

eTable 1. Characteristics of 127,156 VHA Enrollees Age 65+ Hospitalized for COVID-19 Between March 2020 and December 2021 Before Exclusions, by Hospital Type and Payer
eTable 2. Characteristics of Excluded and Included Patients
eTable 3. Characteristics of 52,933 Patients Surviving to Hospital Discharge and Included in Readmission Analyses, by Hospital Type
eTable 4. Multivariable Logistic Regression to Estimate Propensity for VHA Hospital Admission (Odds Ratios for VHA Admission Referent to Community)
eFigure. Distributions of Propensity for VHA Admission (i.e., Treatment Weights) Among Patients Admitted to VHA and Community Hospitals and Approach to Trimming of Weights (Shaded Area Included in Analyses Following Trimming of Outlying Weights, See Text)
eTable 5. Characteristics of VHA-Medicare Dual Enrollees Age 65+ Hospitalized for COVID-19, by Hospital Admission in VHA vs Community, Before and After Inverse Probability of Treatment Weighting (IPTW)

This supplementary material has been provided by the authors to give readers additional information about their work.

© 2023 Ohl ME et al. *JAMA Network Open.*

eTable 1. Characteristics of 127,156 VHA Enrollees Age 65+ Hospitalized for COVID-19 Between March 2020 and December 2021 Before Exclusions, by Hospital Type and Payer

	VHA Hospital	Community Hospital Medicare	Community Hospital CITC
	n=24,938 (19.6%)	n=81,735 (64.3%)	n=20,483 (16.1%)
Age, n (%)			
65-69,	4,649 (18.6)	9,456 (11.6)	3,452 (16.9)
70-74,	8,522 (34.2)	19,712 (24.1)	7,621 (37.2)
75-79,	4,901 (19.7)	15,742 (19.3)	4,275 (20.9)
80-84,	2,743 (11.0)	12,911 (15.8)	2,203 (10.8)
85-90	2,313 (9.3)	12,994 (15.9)	1,748 (8.5)
90+	1,810 (7.3)	10,920 (13.4)	1,184 (5.8)
Sex, male	24,302 (97.5)	73,559 (90.0)	20,046 (97.9)
Race / ethnicity			
White, not Hispanic	16,643 (66.6)	67,324 (82.4)	16,723 (81.6)
Black, not Hispanic	5,525 (22.2)	8,438 (10.3)	1,946 (9.5)
Hispanic	2,141 (8.6)	3,522 (4.3)	1,083 (5.3)
Other, non-white	586 (2.4)	2,334 (2.9)	540 (2.6)
Unknown/missing	43 (0.2)	117 (0.1)	191 (0.9)
Residence			
urban	17,272 (69.3)	50,652 (62.0)	9,869 (48.2)
rural	6,793 (27.2)	26,999 (33.1)	9,024 (44.1)
highly rural	873 (3.5)	4,002 (4.9)	1,581 (7.7)
High social vulnerability census tract	2,697 (11.0)	4,390 (5.5)	1,465 (7.2)
Admission month			
March-Aug.2020	4,158 (16.7)	13,217 (16.2)	1,819 (8.9)
Sep.2020 – Feb 21	11,395 (45.7)	42,952 (52.6)	9,012 (44.0)
March 21-Aug 21	3,913 (15.7)	11,417 (14.0)	3,648 (17.8)
Sept 21 – Dec 21	5,472 (21.9)	14,149 (17.3)	6,004 (29.3)
Gagne comorbidity index, median (IQR)	4.0 (2.0-7.0)	5.0 (3.0-8.0)	4.0 (2.0-7.0)
Transfer in	1,576 (6.3)	8,910 (10.9)	2,138 (10.4)
Mechanical ventilation on day of admission	787 (3.2)	2,361 (2.9)	631 (3.1)
Distance to nearest VHA hospital, miles, median (IQR)	21.4 (9.8-5.5)	61.5 (26.2-109.0)	81.4 (44.8-129.5)

© 2023 Ohl ME et al. *JAMA Network Open.*

Distance to nearest community hospital, miles, median (IQR)	5.6 (2.9-10.7)	6.1 (3.2-14.3)	6.9 (3.2-14.3)
30-day mortality	4,428 (17.7)	22,739 (27.8)	5,215 (25.5)
30-day readmission	6,737 (32.1)	12,649 (19.2)	3,477 (21.0)

All rows n (%) unless otherwise noted.

© 2023 Ohl ME et al. *JAMA Network Open.*

eTable 2. Characteristics of Excluded and Included Patients

	Included patients	Excluded patients
	n=64,856	n=62,300
Admitting hospital, n(%)		
VHA	17035(26.3)	7903(12.7)
Community	47821(73.7)	54397(87.3)
Age, years, mean (SD)	77.6	78.6
Age, years, n (%)		
65-69	8787(13.6)	8778(6.9)
70-74	20315(31.3)	15540(24.9)
75-79	12977(20.0)	11941(19.2)
80-84	8379(12.9)	9478(7.5)
85-90	8125(12.5)	8930(14.3)
90+	6273(9.7)	7641(12.3)
Sex, n(%)		
Male	63562(98.0)	54345(87.2)
Female	1294(2.0)	7950(12.8)
Race / ethnicity, n(%)		
White, not Hispanic	51017(78.7)	49673(79.7)
Black, not Hispanic	8791(13.6)	7118(11.4)
Hispanic	3349(5.2)	3397(5.5)
Other, non-white	1670(2.6)	1790(2.9)
Unknown/missing	29(0.1)	322(0.5)
30-day mortality, n(%)		
Overall	15972(24.6)	16410(26.3)
VHA[1]	3021(17.7)	1407(17.8)
Community[2]	12951(27.1)	15003(27.6)

[1] Denominator is patients admitted to VHA hosptial

[2] Denominator is patients admitted to community hospital

Note: Comorbidity data are incomplete for excluded patients without prior VHA care.

© 2023 Ohl ME et al. *JAMA Network Open.*

eTable 3. Characteristics of 52,933 Patients Surviving to Hospital Discharge and Included in Readmission Analyses, by Hospital Type

	VHA[1] Hospital	Community Hospital
	n=14357(27.1)	n=38576(72.9)
Age, mean (SD)	75.5(7.3)	77.9(7.9)
Age, years, n (%)		
65-69	2832(19.8)	4802(12.5)
70-74	5158(35.9)	11805(30.5)
75-79	2876(20.0)	7797(20.2)
80-84	1490(10.4)	5210(13.5)
85-90	1155(8.0)	5079(13.2)
90+	846(5.9)	3883(10.1)
Sex, n(%)		
Male	13972(97.3)	37820(98.0)
Female	385(2.7)	756(2.0)
Race / ethnicity, n(%)		
White, not Hispanic	9883(68.8)	31708(82.2)
Black, not Hispanic	3082(21.5)	4261(11.1)
Hispanic	1019(7.1)	1623(4.2)
Other, non-white	368(2.6)	963(2.5)
Unknown/missing	5(0.0)	21(0.1)
Residence, n(%)		
Urban	9733(67.8)	20963(54.4)
Rural	4077(28.4)	15104(39.2)
highly rural	547(3.8)	2506(6.5)
High social vulnerability census tract, n(%)	1482(10.3)	2397(6.2)
Admission month, n(%)		
March-Aug.2020	2342(16.3)	5091(13.2)
Sep.2020 – Feb 21	6688(46.6)	20014(51.9)
March 21-Aug 21	2212(15.4)	5748(14.9)
Sept 21 – Dec 21	3115(21.7)	7723(20.0)
Gagne comorbidity index, mean (SD)	5.2 (3.7)	5.4(3.7)
Comorbidities, n(%)		
Hypertension	11778(82.0)	33299(86.3)
Congest Heart Failure	3822(26.6)	11893(30.8)
Arrhythmia	5780(40.3)	17289(44.8)
Myocardial infarction	1781(12.4)	5911(15.3)
Chronic lung disease	5366(37.4)	15302(39.7)
Obesity	3531(24.6)	10768(27.9)
Stroke	2816(19.6)	9372(24.3)
Dementia	2409(16.8)	6222(16.1)
Liver Disease	1413(9.8)	3314(8.6)

© 2023 Ohl ME et al. *JAMA Network Open.*

Diabetes	7410(51.6)	19743(51.2)
Cancer	2764(19.3)	7870(20.4)
Renal Disease	4319(30.1)	13450(34.9)
Depression	4303(30.0)	10701(27.7)
Alcohol use condition	1199(8.4)	2026(5.3)
Drug use condition	720(5.0)	1311(3.4)
Transfer in, n(%)	955(6.7)	3799(9.9)
Mechanical ventilation on admission, n(%)	56(0.4)	514(1.3)
Distance to nearest VHA hospital, miles, mean (SD)	38.0(49.5)	88.7(123)
Distance to nearest community hospital, mean (SD)	8.6(8.7)	9.3(8.8)

[1]VHA Veterans Health Administration

© 2023 Ohl ME et al. *JAMA Network Open.*

eTable 4. Multivariable Logistic Regression to Estimate Propensity for VHA Hospital Admission (Odds Ratios for VHA Admission Referent to Community)

	**Odds ratio (95% CI)**
**Months since March 2020**	**1.013 (1.010 - 1.017)**
**Transfer in**	**1.390 (1.293 - 1.493)**
**Age:** 65-69 vs [age>=90]	**2.271 (2.074 - 2.487)**
70-74 vs [age>=90]	**1.913 (1.763 - 2.075)**
75-79 vs [age>=90]	**1.675 (1.539 - 1.822)**
80-84 vs [age>=90]	**1.297 (1.184 - 1.420)**
85-89 vs [age>=90]	**1.070 (0.975 - 1.174)**
**Race:** BLACK vs WHITE NON HISP	**1.327 (1.254 - 1.405)**
HISPANIC vs WHITE NON HISP	**1.468 (1.350 - 1.597)**
OTHER NON-WHITE vs WHITE NON HISP	**1.190 (1.052 - 1.346)**
UNKNOWN/MISSING vs WHITE NON HISP	**0.691 (0.237 - 2.016)**
**Rural Residence:** Highly rural vs. Urban	**2.124 (1.917 - 2.354)**
Rural vs. Urban	**1.791 (1.699 - 1.888)**
**Comorbidities***	
Congestive heart failure	**1.076 (1.013 - 1.143)**
Valvular disease	**0.608 (0.572 - 0.645)**
Pulmonary circulation disorder	**1.143 (1.062 - 1.231)**
Peripheral vascular disease	**0.725 (0.690 - 0.762)**
Hypertension: non complicated	**0.801 (0.759 - 0.846)**
Complicated	**0.807 (0.764 - 0.853)**
Neurologic disease	**1.072 (1.015 - 1.132)**
Diabetes complicated	**0.930 (0.876 - 0.988)**
Hypothyroidism	**0.835 (0.791 - 0.881)**
HIV AIDS	**1.625 (1.182 - 2.233)**
Arthritis	**0.713 (0.652 - 0.780)**
Coagulopathy	**0.845 (0.786 - 0.909)**
Obesity	**0.851 (0.811 - 0.892)**
Weight loss	**0.913 (0.846 - 0.986)**
Fluid disorder	**1.170 (1.112 - 1.231)**
Alcohol use condition	**1.172 (1.081 - 1.271)**
Depression	**1.062 (1.014 - 1.112)**
Cerebrovascular disease	**0.806 (0.766 - 0.849)**
Dementia	**1.109 (1.041 - 1.182)**
Diabetes	**1.438 (1.314 - 1.575)**
Arthritis	**1.114 (1.062 - 1.168)**
**Gagne Comorbidity Score**	**1.025 (1.012 - 1.038)**
**Distance to nearest VA hospital**	
<16.8 miles vs >116 miles	**3.891 (3.090 - 4.898)**
16.8 – 40.1 miles vs >116 miles	**2.462 (1.981 - 3.060)**
40.1 – 72.7 miles vs >116 miles	**1.932 (1.587 - 2.351)**
72.7 – 116 miles vs >116 miles	**1.337 (1.132 - 1.578)**
**VA vs Community hospital distance (Difference)**	
-13.7 – 9.6 vs >106 miles	**5.620 (4.498 - 7.021)**
9.6 – 30.2 vs >106 miles	**3.699 (2.990 - 4.575)**
30.2 – 62.7 miles vs >106 miles	**2.083 (1.712 - 2.533)**
62.7 – 106 miles vs >106 miles	**1.507 (1.276 - 1.780)**
**Social Vulnerability Index (SVI) >90 (high)**	**1.356 (1.262 - 1.457)**
**c-statistic=0.779**	

*Some Quan comorbidity categories included in model are combined in Table 1 in manuscript (e.g., complicated and non-complicated hypertension combined in Table 1)

© 2023 Ohl ME et al. *JAMA Network Open.*

eFigure. Distributions of Propensity for VHA Admission (i.e., Treatment Weights) Among Patients Admitted to VHA and Community Hospitals and Approach to Trimming of Weights (Shaded Area Included in Analyses Following Trimming of Outlying Weights, See Text)

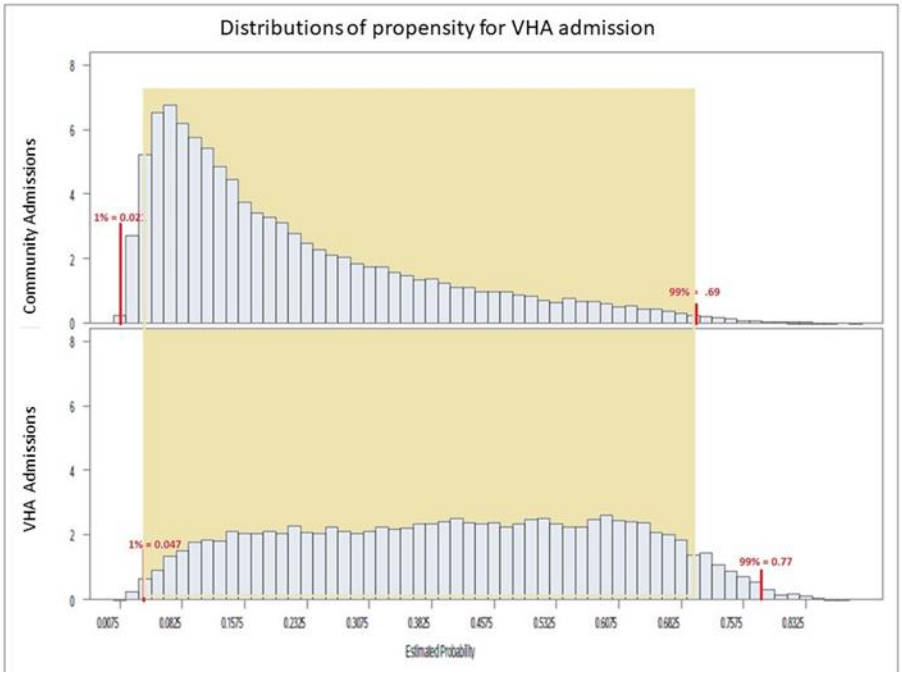

eTable 5. Characteristics of VHA-Medicare Dual Enrollees Age 65+ Hospitalized for COVID-19, by Hospital Admission in VHA vs Community, Before and After Inverse Probability of Treatment Weighting (IPTW) *

	VHA Hospital	Community Hospital	Standardized Difference Before Weighting	Standardized Difference After Weighting
Age, mean (SD)	75.8 (7.9)	79.0 (8.1)	-0.30	-0.01
Age, years, n (%)				
65-69	3,169 (18.60)	3,772 (10.37)	0.19	0.00
70-74	6,018 (35.33)	9,856 (27.11)	0.11	0.00
75-79	3,425 (20.11)	7,152 (19.67)	0.01	0.00
80-84	1,807 (10.61)	5,388 (14.82)	-0.10	0.00
85-90	1,468 (8.62)	5,690 (15.65)	-0.17	-0.01
90+	1,148 (6.74)	4,504 (12.39)	-0.14	0.00
Sex, male, n(%)	16,611 (97.51)	35,716 (98.22)	-0.05	0.01
Race / ethnicity, n(%)				
White, not Hispanic	11,746 (68.95)	29,727 (81.75)	-0.31	-0.01
Black, not Hispanic	3,595 (21.10)	4,147 (11.40)	0.28	0.01
Hispanic	1,249 (7.33)	1,545 (4.25)	0.13	0.00
Other, non-white	440 (2.58)	922 (2.54)	0.00	0.00
Unknown/missing	5 (0.03)	21 (0.06)	-0.01	0.00
Residence, n(%)				
Urban	11,403 (66.94)	20,699 (56.93)	0.26	-0.03
Rural	4,960 (29.12)	13,533 (37.22)	-0.21	0.02
highly rural	672 (3.94)	2,127 (5.85)	-0.11	0.01
High social vulnerability census tract, n(%)	1,762 (10.34)	2,287 (6.29)	0.14	0.00
Admission month, n(%)				
March-Aug.2020	2,858 (16.78)	5,571 (15.32)	0.08	0.05
Sep.2020 – Feb 21	7,890 (46.32)	19,346 (53.20)	-0.10	-0.04
March 21-Aug 21	2,607 (15.30)	5,024 (13.82)	0.02	0.00
Sept 21 – Dec 21	3,680 (21.60)	6,421 (17.66)	0.04	0.00
Gagne comorbidity index, median (IQR)	5.0 (2.0-8.0)	5.0 (3.0-8.0)	-0.08	0.02
Comorbidities, n(%)				
Hypertension	14,053 (82.49)	32,193 (88.53)	-0.12	0.01
CHF	4,730 (27.77)	12,192 (33.53)	-0.09	0.01
Arrhythmia	7,061 (41.45)	17,575 (48.33)	-0.09	0.00
Myocardial infarction	2,217 (13.01)	5,998 (16.50)	-0.08	0.01
Chronic lung disease	6,453 (37.88)	14,559 (40.04)	-0.04	0.02
Obesity	4,197 (24.64)	10,268 (28.24)	-0.07	0.00

© 2023 Ohl ME et al. *JAMA Network Open.*

Stroke	3,410 (20.02)	9,662 (26.57)	-0.11	0.01
Dementia	2,970 (17.43)	6,565 (18.05)	0.02	0.03
Liver Disease	1,667 (9.79)	3,247 (8.93)	0.04	0.00
Diabetes	8,891 (52.19)	18,918 (52.03)	0.01	0.01
Cancer	3,315 (19.46)	7,916 (21.77)	-0.03	-0.01
Renal Disease	5,382 (31.59)	14,019 (38.55)	-0.10	0.00
Depression	5,101 (29.94)	10,209 (28.08)	0.04	0.02
Alcohol use condition	1,368 (8.03)	1,716 (4.72)	0.12	0.00
Drug use condition	820 (4.81)	1,176 (3.23)	0.07	-0.01
Transfer, n(%)	1,204 (7.07)	4,078 (11.22)	-0.14	0.07
Mechanical ventilation on admission, n(%)	550 (3.23)	1,167 (3.21)	0.00	0.00
Distance to nearest VHA hospital, miles, median (IQR)	22.5 (10.1-53.2)	66.1 (28.9-112.3)	-0.53	-0.04
Distance to nearest community hospital, miles, median (IQR)	5.7 (3.0-11.1)	6.2 (3.0-12.2)	-0.07	0.02

*Patient n differs from Table 1 because of trimming of patients with outlying and non-overlapping weights. See Methods section and figure above

© 2023 Ohl ME et al. *JAMA Network Open.*
