# Supplementary material for: Mortality Among US Veterans Admitted to Community vs Veterans Health Administration Hospitals for COVID-19
Source: JAMA Netw Open. 2023 May 30;6(5):e2315902. doi: 10.1001/jamanetworkopen.2023.15902 (PMC10230320; doi:10.1001/jamanetworkopen.2023.15902)
Supplement: Supplement 2. — Data Sharing Statement [file jamanetwopen-e2315902-s002.pdf]

Data Sharing Statement

Ohl. Mortality Among US Veterans Admitted to Community vs Veterans Health Administration Hospitals for COVID-19. *JAMA Netw Open*. Published May 30, 2023.
doi:10.1001/jamanetworkopen.2023.15902

Data
Data available: No

Additional Information
Explanation for why data not available: Access to individual patient data in the Veterans Health Administration (VHA) Corporate Data Warehouse (CDW) is controlled by VHA. We will share aggregate results and code used to generate results according to VHA policy.
